# Quality improvement of childbirth care (Adequate Birth Project) and the assessment of women’s birth experience in Brazil: a structural equation modelling of a cross-sectional research

**DOI:** 10.1186/s12978-022-01536-1

**Published:** 2022-12-15

**Authors:** Mariza Miranda Theme Filha, Tatiana Henriques Leite, Marcia Leonardi Baldisserotto, Ana Paula Esteves-Pereira, Maria do Carmo Leal

**Affiliations:** Department of Epidemiology and Quantitative Methods on Health, National School of Public Health, Oswaldo Cruz Foundation, Rio de Janeiro, Brazil

**Keywords:** Birth experience, Parturition, Assessment of childbirth care, Structural equation models

## Abstract

**Background:**

Brazil’s maternity care is highly medicalized, and obstetric interventions in labour and birth are high, mainly in private health system. The Adequate Birth Project (PPA—Projeto Parto Adequado) is quality improvement project designed to reduce unnecessary caesarian section rates in private hospitals in Brazil. This study evaluated the association between the participation of the PPA and the birth experience assessed by the women.

**Methods:**

It was carried out in 2017/2018 a hospital-based research with a convenience sample of 12 private hospitals among the 23 participants of the project. In this article, a sub-sample of 2348 mothers of 4878 postpartum women, including only women who desired vaginal birth at the ending of pregnancy was analyzed. Multigroup structural equation modelling was used for data analysis to compare vaginal birth and caesarean section. The latent variable was constructed from four items: participation in decisions, respectful treatment during labour and birth, satisfaction with the care during childbirth, satisfaction with care of the baby.

**Results:**

In the vaginal birth group, women who participated in PPA rated the birth experience better than women who did not participate (standardized coefficient: 0.388, p-value: 0.028). On the other hand, this effect was not observed (standardized coefficient: − 0.271, p-value: 0.085) in the caesarean section. Besides, the explicative models for a good birth experience varied to the type of childbirth. Among women with vaginal birth, complication during pregnancy and younger age were associated with a more positive birth experience. In contrast, for women with a caesarean section, access to information and participation in the pregnant group was associated with a better evaluation of the birth experience.

**Conclusions:**

The childbirth care model that encourages vaginal delivery and reduces unnecessary caesarean modulates the birth experience according to the type of birth. This study also highlights the importance of perceived control, support, and relationship with the health team shaping women’s experience with labour and delivery. These factors may affect policy, practice, and research on childbirth care.

**Supplementary Information:**

The online version contains supplementary material available at 10.1186/s12978-022-01536-1.

## Background

The birth experience is multidimensional, affected by personal expectations, the amount of support during labour and birth, the quality of the professional–patient relationship, and decision-making participation. The satisfaction with birth is strongly affected by intrapartum care, and it is expected that the model of care provides for the woman and her baby a positive experience. The models of birth care vary considerably across settings, and midwives and obstetricians can share the care during labour and delivery, including the woman herself in the care decisions, with impact on self-assessment of the quality of care by the woman [1].

Maternity care in Brazil is highly medicalized, and obstetric interventions in labour and birth are high, even among low-risk women [2], and the caesarean section rate is almost 60% [3]. This procedure is even higher in private hospitals, where nearly 90% of women gave birth by caesarean section [4]. Among vaginal birth, only 16.2% are cared for by nurses/nurse-midwives or shared with obstetricians [5].

To change this scenario, in 2015 was launched the “Adequate Birth Project” (PPA—Projeto Parto Adequado), a quality improvement project that aims to identify innovative and viable childbirth care model, valuing normal birth and reducing the percentage of caesarean sections without clinical indication in the private supplementary health system. The PPA has four components: governance, women’s participation, monitoring of indicators, and reorganization of structure and care processes. In each of the drivers, several activities were defined to be implemented by the hospitals that joined the program. For example, in the governance component, the hospital was encouraged to train the health team according to the best evidence available to conduct birth and improve maternal and childcare, besides implementing a financial bonus strategy to implement protocols, routines, and others. In the women’s participation component, women were encouraged to participate in antenatal groups, get secure information, do a birth plan, receive information about best labour practices, visit the hospital, and others. Additionally, hospitals were encouraged to inform the health team perinatal indicators outcomes as CS rate, CS rate by Robson group, childbirth care by nurse-midwives, vaginal birth with episiotomy, admission to Neonatal Intensive Care Unit, and proportion of early-term births (37–38 gestational weeks) and realize meeting to discuss how to improve the outcomes. And finally, to promote doctors and nurses-midwives working collaboratively in labour and birth care and a supportive environment for a vaginal birth. All these drives intend to achieve six outcomes: increase support for the woman giving vaginal birth, increase informed choice for women, improve the quality of childbirth care, increase the proportion of spontaneous or induced labour, increase the rate of full-term birth (≥ 39 weeks), and increase birth experience [6].

In the process of evaluation of the quality of childbirth care offered, one component very important is the women’s assessment of the assistance received. This factor has been considered crucial information for policymakers, managers, and health professionals involved in childbirth care. Besides, there is a growing worldwide trend among policymakers and health managers to pay attention, not only to clinical outcomes but also to the opinion of users [7, 8].

However, there is no consensus in the literature on how the process of evaluation of the care received by puerperal during childbirth occurs. Several models have already been proposed, but they explain little or nothing about how this evaluation mechanism occurs [9, 10]. Even facing this difficulty of definition, studies show that different models of care generate different degrees of satisfaction [11, 12].

In a national study, birth in Brazil, the positive assessment of the care received by women during childbirth was associated with the presence of a companion, privacy in the birthing place, time available to ask questions, clarity of information received, and empathic support from caregivers during labour and birth. This study highlights the importance of the relationship between the woman and the caregivers during labour and birth [13].

Among the many aspects of improving the quality of care proposed by PPA Project, this article aimed to evaluate, precisely, the association between exposition to PPA and the assessment by women of the birth experience, according to type of birth. We hypothesize that women exposed to PPA and vaginal birth present different birth experience compared with women with usual care and caesarean.

## Methods

### The PPA project: study design, sample size, inclusion criteria and data collection

Initially, 23 private hospitals were invited to participate in the PPA project in Brazil. A cross-sectional study was carried out in a convenience sample of 12 hospitals among these 23 hospitals to analyze the project's outcomes. For the sample selection, we considered three criteria that could have affected the degree of implementation of the PPA: hospital location according to geographic macro-region; type of hospital (hospitals owned or not owned by health insurance companies); hospital performance (hospitals that reported good and bad results in achieving the PPA c-section goals, according to administrative data provided by the PPA coordination board). The profile of these hospitals is presented in the Additional file 1. The study was conducted from March to August 2017, 6 to 8 months after the implementation of the PPA. It was eligible all women admitted for the birth of a live newborn (of any gestational age and birth weight) or a stillbirth (with gestational age ≥ 22 weeks and/or birth weight ≥ 500 g). Exclusion criteria were women who gave birth before admission to the hospital; women with extreme communicating difficulty, such as foreigners who could not understand Portuguese; deaf-mute women; women with mental or neurological diseases with severe cognitive impairment; and women who legally interrupted pregnancy. In each hospital, women were invited to participate in the study consecutively, until reaching the planned sample in each hospital.

Face-to-face interview with women at least 6 h after vaginal birth and 12 h after caesarean section was realized, using a structured questionnaire containing maternal identification, socioeconomic condition, previous obstetric history, maternal anthropometric data, prenatal care, illnesses and medication during gestation, labour, and birth, and assessment of care received by the woman and newborn. Also, data from medical records of the women and neonates following their discharge from the hospital, including prenatal cards and ultrasound exams was extracted. Trained interviewers by the study coordination applied all the questionnaires of the research. More information about PPA project can be seen in Torres et al. [6].

### Birth experience study among woman of PPA Project: sample size, inclusion criteria and data analysis

To evaluate the association between exposition to PPA and the assessment by women of the birth experience we conducted an analysis in a sub-sample of PPA project. For this analysis, only women who desired vaginal delivery at the ending of pregnancy were included, totalizing 2348 participants out of 4798 of the total sample. This information was collected during the interview through the answer to two questions:

1. “At the beginning of the pregnancy, what type of delivery did you want to have?” with options of response: vaginal or caesarean.
2. “During the pregnancy, has your preference regarding the type of delivery changed? with options of response: Yes or No.

It was considered “women who desire vaginal birth at the end of pregnancy”: women who want vaginal birth at the beginning and not change their option or women who wish caesarean at the beginning of pregnancy and changed their opinion. Women who opted for scheduled caesarean section (c-section before labour) at the end of pregnancy were excluded (2530 women). This strategy aims to select women with a chance to be exposed to the PPA project.

### Independent variable

Adequate Childbirth Project (PPA): It was considered exposed to PPA all women exposed to the four drives of PPA that stimulated vaginal birth, increasing the proportion of spontaneous or induced labour of full-term birth (≥ 39 weeks) and filled the target population criteria defined by each hospital. In two hospitals, the target population was composed by all primiparous women, in two hospitals by women in Robson’s groups 1 to 4, and in eight hospitals by women admitted by the hospital’s on-call staff (one of which was limited to women in Robson’s group 1 to 4 and another to women without anterior uterine scarring). These criteria were based on the higher probability of vaginal birth. The women that did not fill these criteria received the “Usual Care” service. All these pieces of information were collected in the interview with the women.

### Outcome variable

Birth experience—The latent variable is composed by four indicators:

1. Respectful treatment assessed through the question: “When you are in the hospital for delivery, how do you assess the respect of the professionals when receiving you and speaking to you?” with five options of response: Excellent, Good, Fair, Bad, Terrible.
2. Participation in decisions assessed through the question: “When you are in the hospital for delivery, how do you assess the possibility of participating together with the health team in decisions about your labour and delivery?” with five options of response: Excellent, Good, Fair, Bad, Terrible.
3. Assessment of childbirth care through the question: “In your opinion, the attendance to your delivery was…” with five options of response: Excellent, Good, Fair, Bad, Terrible.
4. Assessment of baby care through the question: “In your opinion, baby care at the maternity ward where he/she was born was…” with five options of response: Excellent, Good, Fair, Bad, Terrible.

All indicators were used in statistical analysis in two categories Excellent/Good and; Fair/Bad/Terrible.

### Other variables of the model

Education (complete high school and complete or incomplete higher education).

Age (14 to 19, 20 to 34, and 35 or more years old).

The economic status of the women was identified using the Brazilian Economic Classification Criteria that encompasses information about the level of education of the household's main breadwinner, the possession of selected appliances and durable assets, and whether there is a domestic employee at home, divided in six categories: A, B1, B2, C1, C2, and D [14]. In the descriptive analysis, the variable was grouped in three categories: A (represented by the richest women), B (represented by intermediate economic status), and C/D (represented the poorest women in the sample).

Parity, divided into two categories, primiparous or multiparous.

Planned pregnancy was evaluated by the question: “When you get pregnant you…” with the following answer options: wanted to become pregnant at that time, wanted to be pregnant later, or did not want to be pregnant. In this analysis, the variable was grouped in “yes” if wanted to become pregnant at that time; and “No” if wanted to be pregnant later or did not want to be pregnant.

Preference of type of birth in the early pregnancy was evaluated by the question: “At the beginning of the pregnancy, what type of delivery did you want to have?” categorized as vaginal or caesarean.

Pregnancy complication indicator was constructed by reference to at least one of the following manifestations during the pregnancy which could influence the health team and the woman to choose a caesarean section: hypertensive syndrome, gestational diabetes, infections, placenta previa, placental abruption, oligodramnia, polydramnia, and restricted uterine growth, analyzed as a dichotomic variable “Yes or No”.

Type of birth categorized as a vaginal birth (including forceps and vacuum-assisted deliveries) or a caesarean section.

Access to information was obtained asking for the woman if she was informed during the prenatal care about how labour begins, risk signs in pregnancy that should make her seek a health service, things she could do during the labour to facilitate the birth of the baby, not cutting the umbilical cord immediately after birth, having skin-to-skin contact with the baby in the delivery, and about breastfeeding in the first hour of life. The variable was a sum of the six items described above varying from 0 to 6. In the descriptive analysis, the variable was aggregate into two categories: less than three pieces of information and four or more information.

Oriented to look for this hospital/maternity because of PPA, with the option “Yes or No”.

### Theoretical model

**Fig. 1:**
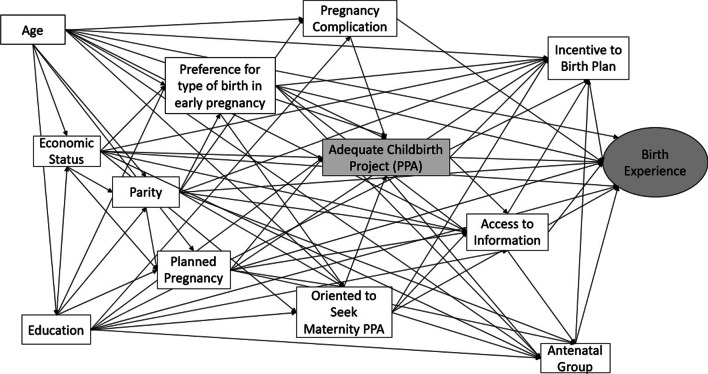
Theoretical model of the association of PPA on birth experience. Adequate Birth Project, Brazil, 2017/2018

Figure 1 shows the theoretical model examining the association between exposition to Adequate Childbirth Project (PPA) (independent variable) and Birth experience (main outcome). The birth experience was defined as a latent variable composed by four indicators: respectful treatment, participation in decisions, assessment of childbirth, and satisfaction with baby care. Other variables which compose the model were considered confounding and mediating based on the existing literature. Observed variables are represented by rectangles, while ellipses represent a latent variable. The theoretical model took into account the temporality of the information obtained in the questionnaire, therefore time runs from left to right. All variables are connected by arrows forming a causal network of information.

### Statistical analysis

Descriptive and bivariate analyses were conducted comparing the “Exposed to PPA” group versus “Usual Care” separately for women who had a vaginal delivery and caesarean section. To compare frequencies between groups, the Chi-square test was used, considering a confidence interval of 95%.

To account for the different number of births per year in each hospital, it was applying a weight to control this disparity. Hospitals with more births per year had a larger weight in sample size. Besides that, each hospital was considered as a stratum. After applying the calibration process described above was performed analysis using structural equation models. To construct the latent variable was considered a factor loading greater than 0.4, with a p-value of less than 0.05, indicative of a good correlation between the observed variable and the construct of interest [15]. Also, multigroup modelling to assess the differences in causal paths between vaginal birth and caesarean section was carried out.

The weighted least squares estimator adjusted by the mean and variance (Weighted Least Squares Mean and Variance adjusted—WLSMV) with probit link and theta parameterization to estimate the coefficients of the model was used. The full information method was used to leading with loss of information in some variables.

To assess suggestions for changes in the initial hypotheses were calculated the modification indices, using the MODINDICES command. When the proposed modifications (modification rates greater than 10) were considered plausible from a theoretical point of view, a new model was developed. In all analyses, the path was significant when the p-value was less than or equal to 0.05 [15, 16].

To assess the model adjustments, three criteria were adopted: The Root Mean Square Error of Approximation (RMSEA), the Comparative Fit Index (CFI) and the Tucker–Lewis Index (TLI). To indicate good fit model, were considered values less than 0.06 to RMSEA and above 0.95 to CFI and TLI [17, 18]. Also, for the RMSEA, the 90% confidence interval (CI) was calculated, and a lower limit close to 0 and the upper limit below 0.08 was considered appropriate [18]. Both the CFI and the RMSEA are sensitive to the lack of model specification and are affected only slightly by the sample size [15, 16].

For data analysis, R version 4.0.3 software (The R Foundation, Vienna, Austria), Mplus 8 software [19] and Stata 13 were used.

This study followed the STROBE (Strengthening the Reporting of Observational Studies in Epidemiology) recommendations for the reporting of cross-sectional research.

## Results

Table 1 describes the characteristics of the study population according to the type of birth and participation in PPA. The most interviewed women who had vaginal birth were young (72.2% under 35 years old), primiparous (61.7%), were in the intermediate socioeconomic category (50.1%), had high education level (70.6%), have had access to information on labour and delivery during prenatal care (81.5%), preferred vaginal delivery at the beginning of pregnancy (92.8%), and the pregnancy was planned (59.1%). Less than 30% were encouraged to make the birth plan or participated in a group of pregnant women, 14.4% presented pregnancy complications, 11.4% were instructed to know the maternity before birth and only 9.9% had previous caesarean section.

**Table 1:** Characteristics of the study population by type of birth, stratified by usual care and PPA group, Adequate Birth Project, Brazil, 2017/2018

Vaginal				
	Total (n = 937)	Usual care (n = 214)	PPA (n = 723)	p-value
	% (CI 95%)	% (CI 95%)	% (CI 95%)	
Woman's age (n = 937)				
14–19 years	2.6 (1.7–3.7)	1.3 (0.4–4.0)	3.0 (0.2–4.3)	**0.015**
20–34 years	69.6 (65.9–73.0)	62.7 (54.7–70.0)	71.6 (67.5–75.4)	
35 or more years	27.8 (24.5–31.4)	36.0 (28.7–43.9)	25.4 (21.7–29.4)	
Woman’s education (n = 937)				
Complete and incomplete high school	29.3 (26.7–32.0)	27.3 (21.58–33.9)	29.8 (26.8–33.1)	0.497
Complete and incomplete higher education	70.6 (67.9–73.2)	72.7 (66.0–78.4)	70.1 (66.8–73.1)	
Economic status (n = 935)				
A	18.5 (16.3–20.9)	11.2 (7.6–16.2)	20.7 (18.7–23.6)	**0.018**
B	50.1 (47.5–54.7)	54.1 (46.3–61.7)	50.3 (46.1–54.4)	
C/D	30.2 (27.2–33.4)	34.6 (27.5–42.4)	28.9 (25.4–32.7)	
Parity				
Primiparous	61.7 (57.9–65.2)	28.7 (22.7–35.3)	71.4 (57.9–65.2)	**< 0.001**
Multiparous	38.3 (34.7–42.0)	71.3 (64.6–77.2)	28.5 (24.9–32.4)	
Previous caesarean section (n = 925)				
No	90.1 (87.4–92.1)	64.2 (56.3–71.3)	97.7 (96.6–98.4)	**< 0.001**
Yes	9.9 (7.8–12.5)	35.8 (28.6–43.6)	2.3 (1.5–3.4)	
Access to information (n = 937)				
Less than 3 information	18.5 (16.0–21.2)	16.1 (11.6–21.7)	19.3 (16.3–22.5)	0.313
More than 4 information	81.5 (78.7–83.9)	83.9 (78.2–88.3)	80.7 (77.4–83.6)	
Pregnancy complication (n = 937)				
No	85.6 (82.7–88.0)	81.2 (73.9–86.7)	86.9 (83.8–89.5)	0.077
Yes	14.4 (11.9–17.2)	18.8 (13.2–26.0)	13.1 (10.4–16.3)	
Preference type of birth early pregnancy (n = 937)				
No	7.2 (5.5–9.2)	7.4 (4.5–11.7)	7.1(5.3–9.6)	0.922
Yes	92.8 (90.7–94.4)	92.6 (88.2–95.4)	92.8 (90.3–94.6)	
Planned pregnancy (n = 936)				
No	40.9 (37.4–44.5)	43.7 (36.4–51.2)	40.1 (36.1–44.2)	0.416
Yes	59.1 (55.4–62.5)	56.3 (48.8–63.5)	59.9 (48.8–63.5)	
Incentive to birth plan (n = 937)				
No	72.9 (23.9–30.5)	63.5 (55.7–70.5)	75.7 (71.8–79.1)	**0.002**
Yes	27.1 (23.9–30.5)	36.5 (29.4–44.2)	24.3 (20.8–28.1)	
Antenatal group (n = 936)				
No	65.2 (61.6–68.5)	67.9 (60.6–74.4)	64.3 (60.2–68.2)	0.398
Yes	34.8 (31.4–38.3)	32.1 (25.6–39.3)	35.6 (31.7–39.7)	
Oriented to Seek Maternity PPA (n = 937)				
No	88.6 (86.3–90.5)	87.9 (83.6–91.2)	88.9 (86.0–91.0)	0.702
Yes	11.4 (9.4–13.6)	12.1 (8.7–16.3)	11.1 (8.9–13.9)	
Participation in decision (n = 931)				
Excellent/Good	94.9 (92.7–96.4)	94.6 (89.4–97.3)	95.1 (92.4–96.7)	0.847
Fair/Bad/Terrible	5.1 (3.5–7.2)	5.4 (2.7–10.5)	4.9 (3.2–7.5)	
Respectful treatment (n = 934)				
Excellent/Good	96.4 (94.4–97.6)	93.8 (88.8–96.6)	97.1 (94.9–98.3)	0.062
Fair/Bad/Terrible	3.6 (23.8–55.3)	6.2 (3.4–11.1)	2.9 (1.6–5.0)	
Satisfaction with childbirth (n = 934)				
Excellent/Good	97.7 (96.1–98.6)	97.4 (94.0–98.8)	97.8 (95.8–98.8)	0.752
Fair/Bad/Terrible	2.3 (13.7–3.8)	2.6 (1.1–5.9)	2.2 (1.1–4.1)	
Satisfaction with baby care (n = 929)				
Excellent/Good	98.1 (96.5–98.9)	96.8 (92.1–98.7)	98.5 (96.6–99.3)	0.238
Fair/Bad/Terrible	1.9 (1.0–3.4)	3.1 (1.2–7.8)	1.5 (0.7–3.3)	

Caesarean-section				
	Total (n = 1411)	Usual care (n = 533)	PPA (n = 878)	
Woman’s age (n = 1411)				
14–19 years	1.6 (1.0–2.4)	0.3 (0.1–0.11)	2.4 (1.5–3.7)	**0.001**
20–34 years	69.4 (66.5–72.2)	66.4 (61.7–70.7)	71.4 (67.5–74.9)	
35 or more years	29.0 (26.1–31.8)	33.3 (28.9–37.9)	26.2 (22.7–30.0)	
Woman’s education (n = 1411)				
Complete or incomplete high school	23.1 (21.1–25.1)	22.9 (19.5–26.5)	23.2 (20.6–25.9)	0.878
Complete and incomplete higher education	76.9 (74.8–78.8)	77.1 (73.4–80.4)	76.7 (74.0–79.3)	
Economic status (n = 1411)				
A	15.9 (14.0–17.8)	16.6 (13.4–20.1)	15.5 (13.2–17.9)	0.160
B	56.1 (53.1–58.9)	52.5 (47.7–57.2)	58.3 (54.3–62.0)	
C/D	28.0 (25.5–30.6)	30.9 (26.6–35.5)	26.2 (22.9–29.8)	
Parity				
Primipara	72.9 (70.1–75.4)	45.3 (40.7–50.0)	89.7 (87.4–91.4)	**< 0.001**
Multiparous	27.1 (24.5–29.8)	54.7 (50.0–59.2)	10.3 (8.5–12.5)	
Previous caesarean section				
No	80.0 (77.4–82.3)	55.5 (50.7–60.2)	94.9 (93.5–96.0)	**< 0.001**
Yes	20.0 (17.6–22.5)	44.5 (39.7–49.2)	8.1 (4.0–6.4)	
Access to information (n = 1411)				
Less than 3 information	22.5 (20.2–24.9)	19.5 (16.0–23.3)	24.3 (21.4–27.5)	0.051
More than 4 information	77.4 (75.1–79.7)	80.5 (76.6–83.9)	75.6 (72.4–78.5)	
Pregnancy complication (n = 1411)				
No	72.2 (69.4–74.8)	68.9 (64.1–73.2)	74.3 (70.8–77.5)	0.055
Yes	27.7 (25.1–30.5)	31.1 (26.7–35.8)	25.7 (22.4–29.1)	
Preference type of birth early pregnancy (n = 1411)				
No	9.1 (7.4–11.0)	9.1 (6.7–12.2)	9.0 (7.0–11.6)	0.972
Yes	90.9 (92.5–92.5)	90.9 (87.7–93.2)	91.0 (88.3–92.9)	
Planned pregnancy (n = 1411)				
No	35.1 (32.3–37.9)	36.8 (32.3–41.4)	34.1 (30.6–37.7)	0.369
Yes	64.8 (62.0–67.6)	63.2 (58.5–67.6)	65.9 (62.2–69.3)	
Incentive to birth plan (n = 1411)				
No	82.3 (79.8–84.5)	83.0 (79.1–86.2)	81.9 (78.6–84.8)	0.686
Yes	17.6 (15.4–20.1)	17.0 (13.7–20.9)	18.0 (15.1–21.3)	
Antenatal group (n = 1411)				
No	64.4 (61.4–67.3)	66.6 (62.0–70.8)	63.1 (59.1–66.8)	0.245
Yes	35.6 (32.7–38.5)	33.4 (29.1–37.9)	36.9 (33.1–40.8)	
Oriented to Seek Maternity PPA (n = 1410)				
No	91.0 (89.2–92.5)	89.7 (86.7–92.0)	91.8 (89.5–93.6)	0.197
Yes	9.0 (7.4–10.7)	10.3 (7.9–13.2)	8.2 (6.3–10.4)	
Moment that Cesarean was decided				
Antenatal care	40.3 (37.3–43.3)	55.4 (50.5–60.1)	31.1 (27.5–34.9)	**< 0.001**
During hospitalization due	10.6 (8.9–12.6)	9.4 (6.8–12.8)	11.3 (9.2–13.8)	
Complication	14.9 (12.8–17.3)	13.1 (9.9–17.0)	16.1 (13.4–19.2)	
Admission at delivery room	34.1 (31.2–37.1)	22.0 (18.3–26.3)	41.4 (37.5–45.4)	
Participation in decision (n = 1402)				
No	94.0 (92.5–95.1)	94.1 (91.4–95.9)	94.0 (92.5–95.1)	0.917
Yes	6.0 (4.8–7.4)	5.9 (4.0–8.5)	6.0 (4.8–7.4)	
Respectful treatment (n = 1405)				
Excellent/Good	96.5 (95.3–97.3)	97.0 (95.2–98.2)	96.1 (94.4–97.3)	0.384
Fair/Bad/Terrible	3.5 (2.6–4.6)	3.0 (1.7–4.8)	3.9 (2.6–5.5)	
Satisfaction with childbirth (n = 1405)				
Excellent/Good	96.9 (95.8–97.7)	97.3 (95.3–98.4)	96.9 (95.2–97.7)	0.579
Fair/Bad/Terrible	3.0 (2.2–4.1)	2.7 (1.5–4.6)	3.1 (2.2–4.7)	
Satisfaction with baby care (n = 1398)				
Excellent/Good	97.9 (96.9–98.5)	97.4 (95.7–98.4)	98.2 (96.8–99.0)	0.336
Fair/Bad/Terrible	2.1 (1.4–3.1)	2.6 (1.5–4.3)	1.8 (0.1–3.1)	

Bold indicates the significant values p-value < 0.05

We observed a very similar profile among women who had a caesarean section, and differently from vaginal birth, it was observed high proportion of complications during pregnancy and childbirth (27.7%), 20% had previous c-section and 59.7% of caesarean sections were decided due to complication, at admission or delivery room. It is worth mentioning that among women who had a vaginal delivery, 35% had a previous cesarean section, being, therefore, vaginal birth after caesarean (VBAC). Among women who had a cesarean section, in 55% of women this was the first cesarean. Regardless of the type of delivery, more than 90% rated participation in decisions, respectful treatment, satisfaction with the childbirth and the treatment received by the baby as excellent.

In the stratified analysis, women who had a vaginal birth or caesarean section exposed to PPA presented similar profiles, and both groups differed significantly from those not exposed to PPA. Women “Exposed to PPA” compared with “usual care” were younger, had high socioeconomic level, were mostly primiparous, had no incentive to perform a birth plan, and had low proportion of previous c-section. Among women who had c-section exposed to PPA, 31.1% of the procedure was decided in the prenatal period, while among those not exposed to PPA the option for c-section was decided during prenatal care in 55.4%.

The results of multigroup structural equation modelling for vaginal births and caesarean sections are shown in Table 2. The global fit estimators of the final model were satisfactory (RMSAEA = 0.012, and CFI = 0.995 and TLI = 0.987) and the latent variable (Birth Experience) showed factor loadings greater than 0.4 in both groups, vaginal births and caesarean.

**Table 2:** Standardized coefficients, standard error, and p-value of the direct effect of Adequate Childbirth Project (PPA) in Birth Experience. Brazil, 2017/2018

Model adjustment	RMSEA			0.012 (0.000–0.021)		
	CFI			0.995		
	TLI			0.987		
	Vaginal (N = 937)			Caesarean (N = 1411)		
	Standardized coefficient	Standard error	p-value	Standardized coefficient	Standard error	p-value
Latent variable						
Birth experience						
Respectful treatment	0.846	0.075	**< 0.001**	0.818	0.064	**<** **0.001**
Participation in decision	0.899	0.076	**<** **0.001**	0.810	0.062	**<** **0.001**
Satisfaction with childbirth	0.863	0.102	**<** **0.001**	0.716	0.063	**<** **0.001**
Satisfaction with baby care	0.841	0.093	**<** **0.001**	0.707	0.079	**<** **0.001**
Direct effect						
Birth experience						
Adequate Childbirth Project (PPA)	0.388	0.177	**0.028**	− 0.271	0.157	0.085
Access to information	− 0.185	0.150	0.219	0.169	0.073	**0.021**
Pregnancy complication	0.519	0.118	**<** **0.001**	0.041	0.073	0.574
Preference of birth in early pregnancy	0.147	0.199	0.459	− 0.003	0.094	0.978
Planned pregnancy	− 0.039	0.123	0.754	− 0.045	0.087	0.604
Incentive to birth plan pregnant	0.247	0.209	0.238	− 0.264	0.118	**0.026**
Pregnant Group Oriented to Seek Maternity PPA	0.006	0.136	0.964	0.260	0.096	**0.007**
Oriented to Seek Maternity PPA	0.290	0.116	**0.012**	− 0.192	0.106	0.069
Age	− 0.278	0.124	**0.025**	0.164	0.088	0.062
Economic status	0.265	0.195	0.175	0.261	0.107	0.015
Education	− 0.170	0.283	0.548	− 0.367	0.161	0.023
Parity	0.215	0.202	0.287	− 0.399	0.202	**0.048**
Incentive to birth plan						
Adequate Childbirth Project (PPA)	− 0.252	0.109	**0.021**	− 0.085	0.137	0.532
Age	0.075	0.086	0.384	0.020	0.078	0.794
Preference of birth in early pregnancy	− 0.266	0.109	**0.014**	− 0.003	0.080	0.971
Economic status	0.092	0.119	0.437	− 0.041	0.089	0.645
Oriented to Seek Maternity PPA	0.226	0.069	**0.001**	0.043	0.084	0.608
Planned pregnancy	− 0.051	0.078	0.510	0.078	0.070	0.265
Education Oriented to Seek Maternity PPA	− 0.040	0.154	0.794	0.123	0.128	0.336
Access to information	0.382	0.064	**<** **0.001**	0.400	0.053	**<** **0.001**
Pregnant group	0.131	0.074	0.077	0.109	0.070	0.118
Parity	− 0.135	0.129	0.295	− 0.190	0.164	0.247
Access to information						
Adequate Childbirth Project (PPA)	− 0.188	0.081	**0.020**	− 0.210	0.084	**0.012**
Age	− 0.117	0.053	**0.028**	0.010	0.048	0.826
Preference of birth in early pregnancy	− 0.111	0.080	0.163	− 0.088	0.061	0.147
Economic status	0.026	0.090	0.773	0.175	0.054	**0.001**
Oriented to Seek Maternity PPA	0.032	0.073	0.664	0.180	0.060	**0.003**
Planned pregnancy	− 0.292	0.054	**<** **0.001**	− 0.076	0.046	0.100
Education	0.317	0.106	**0.003**	0.160	0.080	**0.047**
Parity	− 0.099	0.084	**0.241**	− 0.230	0.101	**0.022**
Pregnant group						
Adequate childbirth Project (PPA)	− 0.104	0.111	0.349	0.065	0.112	0.562
Age	− 0.054	0.079	0.498	− 0.003	0.064	0.967
Preference of birth in early pregnancy	0.102	0.108	0.345	0.012	0.072	0.873
Economic Status	− 0.142	0.111	0.200	− 0.165	0.082	**0.044**
Oriented to Seek Maternity PPA	0.192	0.078	**0.015**	0.342	0.065	**<** **0.001**
Planned pregnancy	0.068	0.082	0.409	0.090	0.061	0.140
Education	0.183	0.140	0.191	0.238	0.113	**0.035**
Access to information	0.093	0.072	0.194	0.134	0.055	**0.015**
Parity	− 0.299	1.121	0.013	− 0.036	0.135	0.788
Adequate Childbirth Project (PPA)						
Pregnancy complication	− 0.166	0.069	**0.016**	− 0.047	0.052	0.371
Preference of birth in early pregnancy	− 0.157	0.079	**0.046**	− 0.063	0.062	0.312
Planned pregnancy	− 0.081	0.074	0.277	0.015	0.054	0.788
Age	0.100	0.074	0.180	0.167	0.050	**0.001**
Economic status	− 0.120	0.110	0.277	0.078	0.059	0.189
Education	− 0.078	0.144	0.590	− 0.311	0.088	**<** **0.001**
Oriented to Seek Maternity PPA	− 0.120	0.078	0.125	− 0.106	0.060	0.080
Parity	− 0.664	0.067	**<** **0.001**	− 0.852	0.045	**<** **0.001**
Oriented to Seek Maternity PPA						
Preference of birth in early pregnancy	0.093	0.137	0.500	− 0.114	0.085	0.181
Planned pregnancy	− 0.092	0.089	0.303	0.003	0.081	0.970
Age	− 0.107	0.089	0.231	− 0.112	0.074	0.134
Economic status	− 0.231	0.146	0.113	− 0.106	0.106	0.317
Education	0.348	0.161	**0.031**	0.204	0.127	0.109
Parity	− 0.081	0.099	0.413	0.008	0.095	0.931
Pregnancy complication						
Age	0.211	0.078	**0.007**	0.053	0.051	0.305
Education	− 0.041	0.083	0.624	− 0.168	0.057	**0.003**
Parity	− 0.011	0.085	0.900	0.051	0.066	0.436
Preference of birth in early pregnancy						
Age	0.122	0.085	0.152	− 0.014	0.066	0.837
Economic status	0.136	0.142	0.340	− 0.061	0.088	0.485
Education	− 0.447	0.161	**0.006**	0.116	0.106	0.273
Parity	− 0.242	0.091	**0.008**	− 0.047	0.083	0.573
Planned pregnancy						
Age	− 0.292	0.057	**<** **0.001**	− 0.369	0.041	**<** **0.001**
Economic status	− 0.333	0.086	**<** **0.001**	− 0.175	0.059	**0.003**
Education	0.068	0.101	0.500	− 0.040	0.077	0.605
Parity	0.161	0.066	**0.015**	0.173	0.058	**0.003**
Parity						
Economic status	0.102	0.090	0.256	0.192	0.062	**0.002**
Age	0.445	0.050	**<** **0.001**	0.373	0.040	**<** **0.001**
Education	− 0.336	0.106	**0.001**	− 0.504	0.069	**<** **0.001**
Economic status						
Age	0.118	0.036	**0.001**	0.158	0.030	**<** **0.001**
Education	0.667	0.034	**<** **0.001**	0.578	0.028	**<** **0.001**
Education						
Age	0.452	0.032	**<** **0.001**	0.324	0.030	**<** **0.001**

Bold indicates the significant values p-value < 0.05

Table 2 also informs the standardized coefficients of the direct effect of PPA on birth experience considering vaginal birth and caesarean. Considering the main aim, women who participate in PPA shows an increased positive evaluation of the birth experience (standardized coefficient: 0.388, p-value: 0.028) when compared with women in “usual care” group in vaginal births. This effect was not observed in women who had a caesarean (standardized coefficient: − 0.271, p-value: 0.085). Besides that, it was testing seven indirect effects between PPA and assessment of the birth experience. No pathway was shown statistical significance considering vaginal births and caesarean section (see Additional file 1).

Table 2 shows other characteristics that could affect the assessment of birth experience in vaginal births and caesarean section. Considering the group of vaginal births, women who have a complication during pregnancy (standardized coefficient: 0.519, p-value: < 0.001) and younger women (standardized coefficient: − 0.278, p-value: 0.025) were associated with a better assessment of the birth experience. In contrast, other variables were important for women with a caesarean section, among them “access to information” (standardized coefficient: 0.169, p-value: 0.021) and “participation in pregnant group” (standardized coefficient: 0.260, p-value: 0.007) were associated with better evaluation of birth experience. To have an “incentive to a birth plan” (standardized coefficient: − 0.264, p-value: 0.026) and parity (standardized coefficient: − 0.399, p-value: 0.048) were associated negatively with the assessment of birth experience.

**Fig. 2:**
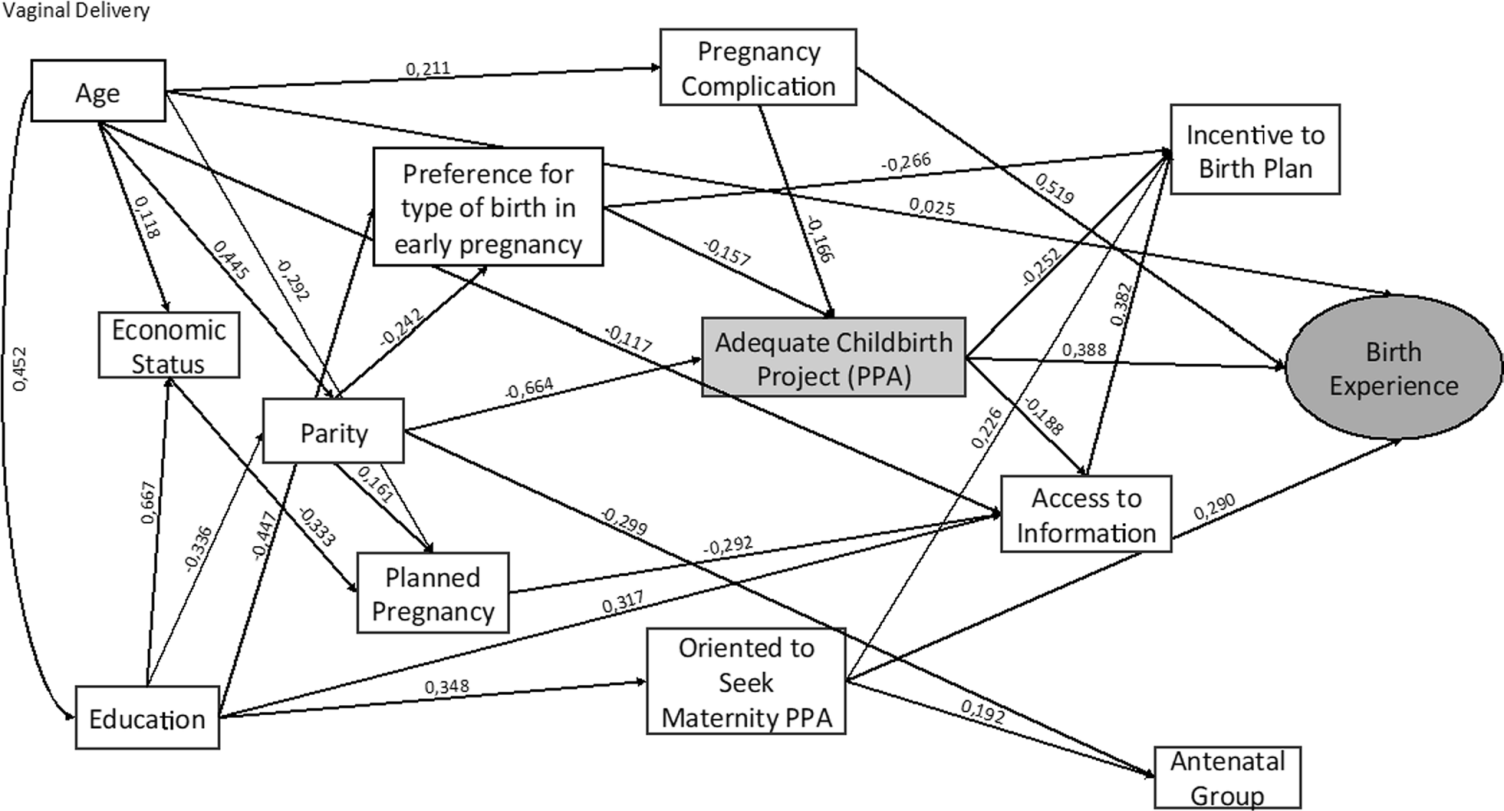
Theoretical model and the standardized coefficients estimated of the association of PPA on birth experience in the vaginal delivery group. Adequate Birth Project, Brazil, 2017/2018

**Fig. 3:**
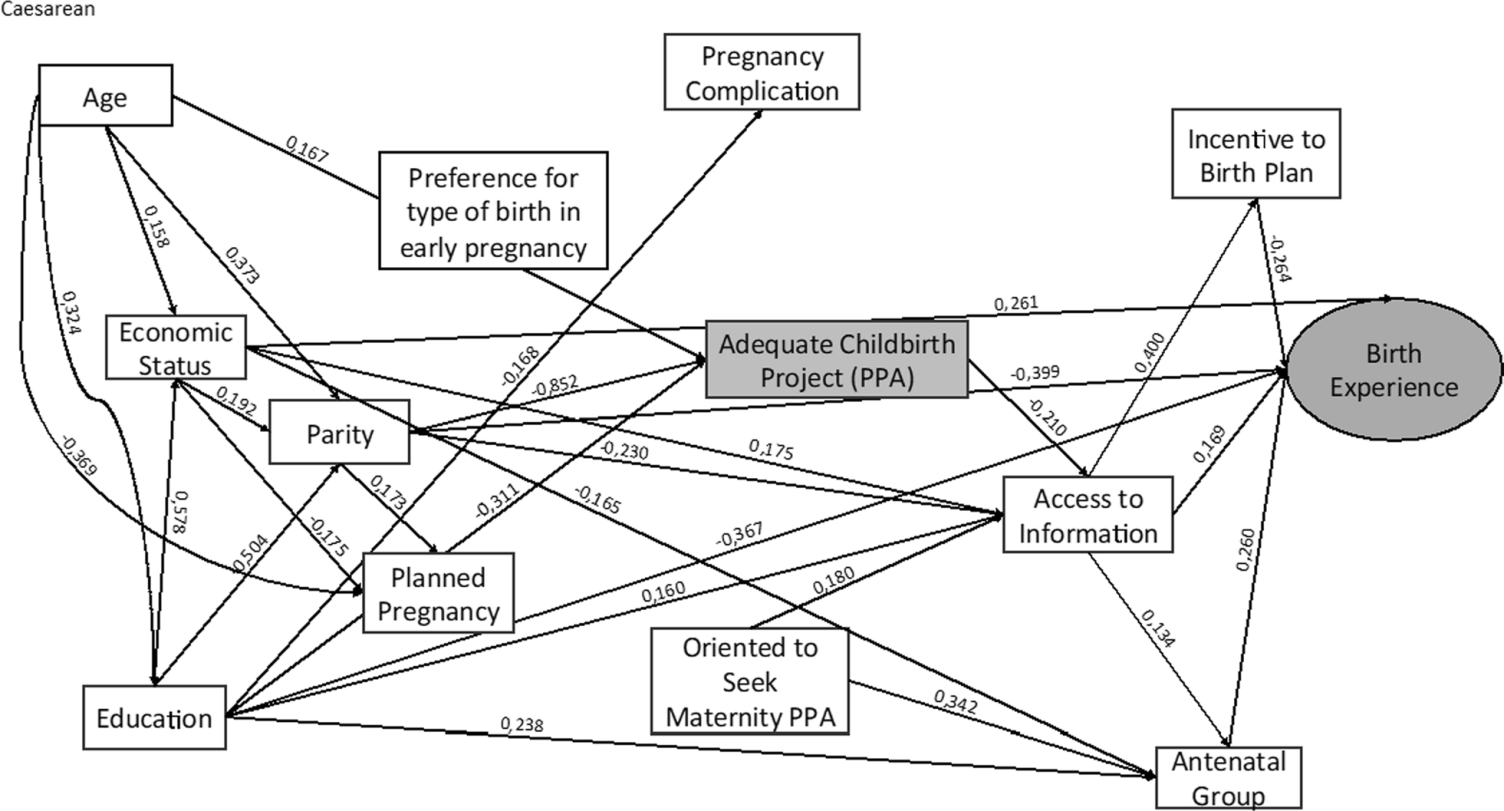
Theoretical model and the standardized coefficients estimated of the association of PPA on birth experience in the Caesarean group. Adequate Birth Project, Brazil, 2017/2018

Figures 2 and 3 show that the statistically significant standardized coefficients that best explain the vaginal delivery model and the cesarean model are different, configuring explanatory models that vary according to the type of delivery.

## Discussion

This study investigated the association between exposition to a quality improvement of childbirth care (PPA) and assessment of birth experience among women who had vaginal birth and caesarean. The results demonstrate that women who participated in PPA showed an increased positive evaluation of the birth experience in the vaginal delivery group. Nevertheless, this effect was not observed in women who had a caesarean. Our results agree with the literature that the type of delivery impacts the subjective experience of childbirth [20–22]. The lack of association of exposure to PPA and birth experience among women submitted to caesarean can be attributable to the high caesarean rate in Brazil. It might have shaped women’s expectations by normalizing this practice, not affecting the birth experience. Another hypothesis, the c-section in the PPA group, was decided at the admission or delivery room (68.9%), suggesting a medical indication for the procedure and its acceptance due to a potential risk for mother and baby. It is also possible that the PPA project had differentiated the care among women in vaginal delivery, stimulating this practice and offering more special attention and support for women.

As mentioned in the introduction, the birth experience is multidimensional and a highly individual experience, affected not only by obstetric factors. Multiple interrelated factors contribute to the construction of the childbirth experience, including perceived control, support, and the relationship with the health team. A systematic review of randomized control trials of interventions in pregnancy or labour identified successful strategies to create a positive perception: supporting women during birth (Risk ratio = 1.35, CI 95% 1.07 to 1.71), intrapartum care with minimal intervention (Risk ratio = 1.29, CI 95% 1.15 to 1.45), and birth preparedness and readiness for complications (Mean Difference = 3.27, 95% confidence interval 0.66 to 5.88). The last one included attending the antenatal group and having a birth plan to prepare women for birth [23]. Other systematic review exploring risk and protective factors for women’s subjective childbirth experience and birth satisfaction showed that the main protective factors were perceived control during labour and satisfaction regarding partner’s support, highlighting the importance of maternal interpersonal and professional relationships [24].

A qualitative study carried out in Australia to evaluate women’s experiences of maternity care after the implementation of maternity care reform identified four broad themes to improving quality of childbirth: quality of care (interpersonal and technical); access to choices and involvement in decision-making; unmet information needs; and dissatisfaction with the care environment [25]. Therefore, support from the social network and health professionals is essential for a positive birth experience, even if the birth was protracted or with medical complications [26].

The factors pointed out in these studies are following our results about the importance of support, respectful treatment, and participation in the decisions in the birth experience. The theoretical model proposed to explain the relationship between PPA and birth experience in this research presented adequate adjustment. All four components of the latent variable could measure birth experience for both vaginal birth and c-section. However, it is worth highlighting that the explicative models for good birth experience are different according to the type of childbirth.

The invariance measurement between groups was observed, indicating that the theoretical model to explain the relationship between PPA and birth experience is different between vaginal delivery and caesarean. This fact shows that the type of birth influences how women evaluate the assistance received for labour and delivery. These results are corroborated by d'Orsi and colleagues [27], who found a direct association between type of delivery and satisfaction with labour and delivery care.

Our study observed a negative association between parity and childbirth experience among the women who had a caesarean section, indicating that the previous birth experience influences future birth experience in this group. In the caesarean group, multiparous women tended to evaluate more negatively the assistance received compared to primiparous women. Domingues and colleagues [28] also verified an association between parity and satisfaction with care received. The authors observed that primiparous women expressed greater satisfaction than multiparous women (p-value = 0.0061). It is possible that multiparous women would be more critical comparing the recent experience with previous experience(s). On the other hand, Brown and Lumley [29] found more dissatisfaction among primiparous women than multiparous. According to the authors, primiparous women tend to have longer, more painful deliveries with more medical intervention and the worst evaluation of labour and childbirth.

A factor that can influence how she evaluates the care received in the maternity is the intentionality of the pregnancy. A planned or unplanned pregnancy can affect the perception of delivery, consequently influencing the degree of satisfaction [12, 28]. However, no direct association between pregnancy planning and birth experience in either group in our study was found. This result could be explained by the sample’s characteristics, which was composed of an extract of the Brazilian population with a higher prevalence of planned pregnancies than the general population [30].

Some studies show that sociodemographic and economic variables (education, race, economic situation) influence the childbirth experience [31, 32]. Leal and colleagues pointed out differences in the degree of satisfaction between black or brown women and white women. Non-white women tend to have a lower degree of satisfaction than white women. Regarding schooling, they found that satisfaction increases with the years of study [31]. However, our research observed no association between socioeconomic variables and birth experience. A possible explanation is the composition of the study population with women with higher education, higher social class, and white skin colour.

## Strengths and limitations

Although this study has presented results consistent with other researches, it is essential to highlight some methodological limitations that may have influenced women's responses. As the interview was conducted in the immediate postpartum period, it is possible the introduction of biases. A critical factor reported in the literature is the tendency for women to assess the care received during labour and childbirth more positively than it was. Van Teijlingen and colleagues [8] called this trend “gratitude bias”. According to these authors, this bias permeates and hinders many studies investigating mothers’ assessment and satisfaction with childbirth care received. These authors suggest that some women cannot negatively rate their care because they consider such an act to be ingratitude for the positive outcome of childbirth. This bias tends to be stronger during hospitalization and decreases over time. Also, studies recommend that this type of question be made after hospital discharge because women may feel embarrassed and afraid of reprisals from the health care team while still in the hospital.

Despite this limitation, evidence-based knowledge about childbirth shows that a positive birth experience is an important goal of obstetric care. Good professional support quality can promote positive women’s feelings. A trusting relationship and good communication are steps to be followed by health providers and health teams to achieve the best care rate for women and babies (see Additional file 2).

## Conclusions

This study highlights women’s experiences of childbirth associated with a new model of quality improvement of childbirth care and reaffirms the importance of improvement of labour and birth care in Brazil. It also provides contextualized information on the importance of women's feelings of respectful treatment, participation in the decision, satisfaction with labour, delivery, and childcare in shaping their birth experience. These factors must be considered in the planning of health policies to improve the quality of obstetric care, as recommended by the World Health Organization [33].

## Supplementary Information

**Additional file 1.** Characteristics of private hospitals included in PPA (Adequate Birth Project). Brazil, 2017/2018.

**Additional file 2.** Standardized coefficients, standard error, and p-value of the indirect effect of PPA (Adequate Birth Project) in Birth Experience. Brazil, 2017/2018.

## Data Availability

The datasets used and/or analyzed during the current study are available from the corresponding author on reasonable request.

## Abbreviations

CFI: Comparative Fit Index
CI: Confidence interval
CS: Caesarean section
PPA: Project “Parto Adequado” (Adequate Birth Project)
RMSEA: Root Mean Square Error of Approximation
STROBE: Strengthening the Reporting of Observational Studies in Epidemiology
TLI: Tucker–Lewis Index
VBAC: Vaginal birth after caesarean
WLSMV: Weighted Least Squares Mean and Variance
