# Correction: Quality improvement of childbirth care (Adequate Birth Project) and the assessment of women’s birth experience in Brazil: a structural equation modelling of a cross-sectional research

**DOI:** 10.1186/s12978-023-01570-7

**Published:** 2023-01-26

**Authors:** Mariza Miranda Theme Filha, Tatiana Henriques Leite, Marcia Leonardi Baldisserotto, Ana Paula Esteves-Pereira, Maria do Carmo Leal

**Affiliations:** Department of Epidemiology and Quantitative Methods On Health, National School of Public Health, Oswaldo Cruz Foundation, Rio de Janeiro, Brazil

**Correction: Reproductive Health (2022) 20:1** https://doi.org/10.1186/s12978-022-01536-1

Following publication of the original article [1], we have been notified that the Funding note should be as follows:

“This work was supported, in part by the Bill & Melinda Gates Foundation [OPP1142172] and DECIT/MoH Brazil/CNPq [401715/2015-1]. Under the grant conditions of the Foundation, a Creative Commons Attribution 4.0 Generic License has already been assigned to the Author Accepted Manuscript version that might arise from this submission”.
