# Supplementary material for: Quality improvement of childbirth care (Adequate Birth Project) and the assessment of women’s birth experience in Brazil: a structural equation modelling of a cross-sectional research
Source: Reprod Health. 2022 Dec 15;20(Suppl 2):1. doi: 10.1186/s12978-022-01536-1 (PMC9756594; doi:10.1186/s12978-022-01536-1)
Supplement: Supplementary file 1 — Additional file 1. Characteristics of private hospitals included in PPA (Adequate Birth Project). Brazil, 2017/2018. [file 12978_2022_1536_MOESM1_ESM.docx]

Additional File 1 – Characteristics of private hospitals included in PPA (Adequate Birth Project). Brazil. 2017/2018.

| Hospital | Region | Hospital owned by health insurance | Number of obstetric beds* | Births/year* | Caesarean rate* |
| --- | --- | --- | --- | --- | --- |
| 1 | Northeast | No | 30 | 1727 | 79.8 |
| 2 | Southeast | No | 14 | 1180 | 89.7 |
| 3 | Southeast | Yes | 20 | 2235 | 79.2 |
| 4 | Southeast | No | 29 | 1687 | 65.5 |
| 5 | South | Yes | 24 | 1457 | 70.6 |
| 6 | South | No | 39 | 4373 | 81.9 |
| 7 | Southeast | No | 22 | 2529 | 73.9 |
| 8 | Southeast | No | 57 | 4663 | 73.5 |
| 9 | Southeast | Yes | 39 | 2518 | 74.6 |
| 10 | Southeast | No | 26 | 2526 | 78.5 |
| 11 | Southeast | No | 95 | 8555 | 78.2 |
| 12 | Southeast | No | 65 | 3929 | 79.3 |

*Year of reference = 2017
