# Supplementary material for: Quality improvement of childbirth care (Adequate Birth Project) and the assessment of women’s birth experience in Brazil: a structural equation modelling of a cross-sectional research
Source: Reprod Health. 2022 Dec 15;20(Suppl 2):1. doi: 10.1186/s12978-022-01536-1 (PMC9756594; doi:10.1186/s12978-022-01536-1)
Supplement: Supplementary file 2 — Additional file 2. Standardized coefficients, standard error, and p-value of the indirect effect of PPA (Adequate Birth Project) in Birth Experience. Brazil, 2017/2018. [file 12978_2022_1536_MOESM2_ESM.docx]

Additional file 2: Standardized coefficients, standard error, and p-value of the indirect effect of PPA (Adequate Birth Project) in Birth Experience. Brazil, 2017/2018.

|  | **Vaginal** (N=937) | | | **Caesarean** (N=1412) | | | |
| --- | --- | --- | --- | --- | --- | --- | --- |
|  | Standardized  Coefficient | Standard  Error | p-value | Standardized  Coefficient | Standard  Error | p-value | |
|  | | | | | | |  |
| **Indirect effect** | | | | | | | |
| Adequate Childbirth Project (PPA)🡪 Incentive to Birth Plan 🡪 Birth Experience | -0.074 | 0.079 | 0.347 | 0.024 | 0.045 | 0.591 | |
| Adequate Childbirth Project (PPA)🡪 Pregnant group 🡪 Birth Experience | -0.001 | 0.017 | 0.964 | 0.018 | 0.036 | 0.616 | |
| Adequate Childbirth Project (PPA)🡪 Access to information 🡪 Birth Experience | 0.041 | 0.041 | 0.316 | -0.038 | 0.036 | 0.294 | |
| Adequate Childbirth Project (PPA)🡪 Pregnant group🡪 Incentive to Birth Plan🡪 Birth Experience | 0.004 | 0.006 | 0.501 | -0.002 | 0.004 | 0.620 | |
| Adequate Childbirth Project (PPA)🡪 Access to information🡪 Incentive to Birth Plan 🡪Birth Experience | -0.021 | 0.021 | 0.318 | 0.024 | 0.024 | 0.322 | |
| Adequate Childbirth Project (PPA)🡪 Access to information 🡪 Pregnant group 🡪 Birth Experience | 0.000 | 0.003 | 0.964 | -0.008 | 0.008 | 0.350 | |
| Adequate Childbirth Project (PPA)🡪 Access to information🡪 Pregnant group 🡪Incentive to Birth Plan🡪 Birth Experience | -0.001 | 0.001 | 0.466 | 0.001 | 0.001 | 0.424 | |
